# Predicting Infectious Disease Using Deep Learning and Big Data

**DOI:** 10.3390/ijerph15081596

**Published:** 2018-07-27

**Authors:** Sangwon Chae, Sungjun Kwon, Donghyun Lee

**Affiliations:** Department of Business Administration, Korea Polytechnic University, 237 Sangidaehak-ro, Siheung-si, Gyeonggi-do 15073, Korea; chaesw1993@kpu.ac.kr (S.C.); solomonseal@kpu.ac.kr (S.K.)

**Keywords:** infectious disease prediction, deep neural network, long short-term memory, deep learning, social media big data

## Abstract

Infectious disease occurs when a person is infected by a pathogen from another person or an animal. It is a problem that causes harm at both individual and macro scales. The Korea Center for Disease Control (KCDC) operates a surveillance system to minimize infectious disease contagions. However, in this system, it is difficult to immediately act against infectious disease because of missing and delayed reports. Moreover, infectious disease trends are not known, which means prediction is not easy. This study predicts infectious diseases by optimizing the parameters of deep learning algorithms while considering big data including social media data. The performance of the deep neural network (DNN) and long-short term memory (LSTM) learning models were compared with the autoregressive integrated moving average (ARIMA) when predicting three infectious diseases one week into the future. The results show that the DNN and LSTM models perform better than ARIMA. When predicting chickenpox, the top-10 DNN and LSTM models improved average performance by 24% and 19%, respectively. The DNN model performed stably and the LSTM model was more accurate when infectious disease was spreading. We believe that this study’s models can help eliminate reporting delays in existing surveillance systems and, therefore, minimize costs to society.

## 1. Introduction

Infectious disease occurs when a person is infected by a pathogen from another person or an animal. It not only harms individuals, but also causes harm on a macro scale and, therefore, is regarded as a social problem [1]. At the Korea Center for Disease Control (KCDC), infectious disease surveillance is a comprehensive process in which information on infectious disease outbreaks and vectors are continuously and systematically collected, analyzed, and interpreted. Moreover, the results are distributed quickly to people who need them to prevent and control infectious disease. The KCDC operates a mandatory surveillance system in which mandatory reports are made without delay to the relevant health center when an infectious disease occurs and it operates a sentinel surveillance system in which the medical organization that has been designated as the sentinel reports to the relevant health center within seven days. The targets of mandatory surveillance consist of a total of 59 infectious diseases from Groups 1 to 4 by the KCDC. The targets of sentinel surveillance include influenza from Group 3 along with 21 infectious diseases from Group 5. Overall, a total of 80 infectious diseases in six groups are monitored. In the current Korean infectious disease reporting system, if there is a legally defined infectious disease patient at a medical organization, a report is made to the managing health center through the infectious disease web reporting system. The managing health center reports to the city and province health offices through another system and the city and province health offices report to the KCDC.

In the conventional reporting system, some medical organizations’ infectious disease reports are incomplete and delays can occur in the reporting system. For instance, in the traditional influenza surveillance system, around two weeks elapses between when a report is made and when it is disseminated [2]. The KCDC has been running an automated infectious disease reporting system as a pilot project since 2015. However, by 2017, only 2.3% of all medical organizations were participating in the pilot project. In medical organizations using the conventional infectious disease reporting system, a large number of missing and delayed reports can occur, which hinders a prompt response to infectious disease. As such, it is necessary to create a data-based infectious disease prediction model to handle situations in real time. Furthermore, if this model can understand the extent of infectious disease trends, the costs to society from infectious disease can be minimized.

An increasing number of researchers recognize these facts and are performing data-based infectious disease surveillance studies to supplement existing systems and design new models [3,4,5,6,7,8,9]. Among these, studies are currently being performed on detecting infectious disease using big data such as Internet search queries [10,11,12,13,14,15]. The Internet search data can be gathered and processed at a speed that is close to real time. According to Towers et al., Internet search data can create surveillance data faster than conventional surveillance systems [16]. For example, when Huang et al. predicted hand, foot, and mouth disease using the generalized additive model (GAM), the model that included search query data obtained the best results. As such, it has been reported that new big data surveillance tools have the advantage of being easy to access and can identify infectious disease trends before official organizations [17]. In addition to Internet search data, social media big data is also being considered. Tenkanen et al. report that social media big data is relatively easy to collect and can be used freely, which means accessibility is satisfactory and the data is created continuously in real time with rich content [18]. As such, studies have used Twitter data to predict the occurrences of mental illness [19] and infectious disease [20,21,22,23] in addition to predictions in a variety of other scientific fields [24,25,26,27]. In particular, a study by Shin et al. reported that infectious diseases and Twitter data are highly correlated. There is the possibility of using digital surveillance systems to monitor infectious disease in the future [20]. When these points are considered, using search query data and social media big data should have a positive effect on infectious disease predictions.

In addition to these studies, there are also studies that have used techniques from the field of deep learning to predict infectious disease [22,23,28,29]. Deep learning is an analysis method and, like big data, it is being actively used in a variety of fields [30]. Deep learning yields satisfactory results when it is used to perform tasks that are difficult for conventional analysis methods [31,32,33]. In a study by Xu et al., a model that used deep learning yielded better prediction performance than the generalized linear model (GLM), the least absolute shrinkage and selection operator (LASSO) model, and the autoregressive integrated moving average (ARIMA) model [28]. As such, methods of predicting infectious disease that use deep learning are helpful for designing effective models.

There are also examples of infectious disease prediction based on environmental factors such as weather [34,35,36,37]. Previous studies have confirmed that weather data comprises a factor that has a great influence on the occurrence of infectious diseases [38,39,40]. Liang et al. showed that rainfall and humidity are risk factors for a hemorrhagic fever with a renal syndrome [41]. In addition, a study by Huang et al. reported that trends in dengue fever show a strong correlation with temperature and humidity [42]. Previous studies indicate that infectious disease can be predicted more effectively if weather variables, Internet big data, and deep learning are used.

Most previous research has attempted to predict infectious disease using Internet search query data alone. However, as discussed above, it is necessary to also consider various big data and environmental factors such as weather when predicting infectious disease. In addition, in the case of models that use deep learning, it is possible to improve prediction performance by optimizing the deep learning model by optimizing its parameters. Therefore, the aim of this study is to design a model that uses the infectious disease occurrence data provided by the KCDC, search query data from search engines that are specialized for South Korea, Twitter social media big data, and weather data such as temperature and humidity. According to a study by Kwon et al., a model that considers the time difference between clinical and non-clinical data can detect infectious disease outbreaks one to two weeks before current surveillance systems [43]. Therefore, this study adds lag to the collected dataset to take temporal characteristics into account. In addition, in the design process, a thorough testing of all the input variable combinations is performed to examine the effects of each resulting dataset on infectious disease outbreaks and select the optimal model with the most explanatory power. The model’s prediction performance is verified by comparing it with an infectious disease prediction model that uses a deep learning method and an infectious disease prediction model that uses time series analysis.

Ultimately, using the results obtained by this study, it should be possible to create a model that can predict trends about the occurrence of infectious disease in real time. Such a model can not only eliminate the reporting time differences in conventional surveillance systems but also minimize the societal costs and economic losses caused by infectious disease.

The remainder of this paper is organized as follows. Section 2 describes the data sources and standards used in this study and introduces the analysis methodology used to design the prediction model. In Section 3, the analysis results are described and their implications are discussed. Section 4 discusses the results. Section 5 concludes the paper.

## 2. Data and Methods

### 2.1. Research Data

As mentioned above, this study uses four kinds of data to predict infectious disease, which includes search query data, social media big data, temperature, and humidity. The standards for the non-clinical data are as follows. Data from 576 days between 1 January, 2016 and 29 July, 2017 was used. The infectious diseases selected for this study are subject to mandatory reporting. Unlike those diseases subject to mandatory reporting, diseases subject to sentinel reporting aggregate data on a weekly basis. Since prediction is also performed on a weekly basis, it is difficult to cope with infectious diseases in real time. Therefore, diseases that are subject to sentinel reporting were excluded from the study. Moreover, the study excluded infectious diseases with an annual occurrence rate of less than 100 as well as infectious diseases that have a statistically insignificant model with an adjusted R-squared value of less than 0.25 when regression analysis is performed using all variables. Three infectious diseases satisfied all conditions, which include malaria, chickenpox, and scarlet fever. The search data was collected from the Naver Data Lab (https://datalab.naver.com/keyword/trendSearch.naver). The usage share data provided by InternetTrend (http://internettrend.co.kr/trendForward.tsp) on search engines in the health/medicine field in the first half of 2017 shows that the Naver search engine had the highest usage share (86.1%) in South Korea. Therefore, it was chosen as the search engine for extracting search data. Note that the collected search data consists of only Korean terms because the search engine is specific to South Korea. The search queries used in this study consisted of the infectious disease’s proper name and symptoms (e.g., “chickenpox” and “chickenpox symptoms” in Korea). The frequency of inquiries using these search queries were used as the search data. The number of searches were normalized with respect to the largest number of searches within the study period.

Weather data (temperature and humidity) were collected from the Korea Meteorological Administration’s weather information open portal (https://data.kma.go.kr). Hourly data collected from weather stations nationwide was converted into daily average data for each station. In Gyeonggi-do province, where around half of South Korea’s population lives, there are many weather stations crowded together. There was a concern that simply finding the averages of the daily data for each station would cause errors to occur, so the following process was performed. First, the averages of the data from each station were collected for the eight provinces in South Korea (Gyeonggi-do, Gangwon-do, Chungcheongnam-do, Chungcheongbuk-do, Jeollanam-do, Jeollabuk-do, Gyeongsangbuk-do, and Gyeongsangnam-do). Next, the averages of the data for each of the eight provinces were found to obtain South Korea’s national average weather data. Average temperature (degrees Celsius) and average humidity (percentage) were recorded.

Social media big data was collected for each infectious disease from Twitter through a web crawler that used the Python Selenium library. For the Twitter data, the daily number of tweets mentioning infectious disease was recorded.

Lastly, infectious disease data was collected from the infectious disease web statistics system (https://is.cdc.go.kr/dstat/index.jsp). This data consists of the daily number of people who were infected throughout South Korea. Table 1 shows the sources and descriptions of the data.

Table 2 shows the statistics for each of the infectious disease variables used in this study. In the case of temperature and humidity, the same conditions were used, which means they were put in a shared category. The data in Table 2 shows that an average of 166.76 people are infected with chickenpox daily with a standard deviation of 98.37 and the daily Naver frequency average is 33.94 with a standard deviation of 15.50. We observed that all the statistics for chickenpox are higher than those for other infectious diseases.

### 2.2. Analysis Method

**Figure 1:**
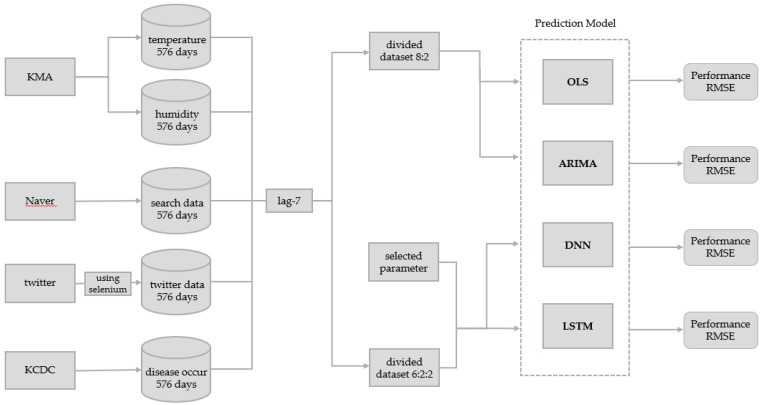
Infectious disease prediction model.

Figure 1 shows the overall framework of the model used in this study including the data collection process and the comparison of models designed using the deep neural network (DNN) method, the long-short term memory (LSTM) method, the autoregressive integrated moving average (ARIMA) method, and the ordinary least squares (OLS) method.

This study constructed an infectious disease surveillance model that uses non-clinical search data, twitter data, and weather data. To design the optimal prediction model, the OLS models that use all possible combinations of variables in the dataset were created. The adjusted R-squared values of each model were compared. In addition, lags of 1–14 days were added to each infectious disease and their adjusted R-squared values were compared in a preliminary analysis. A lag of seven days, which had high explanatory power for all infectious diseases, was selected as the optimal lag parameter. The optimal parameters were used to create the OLS, ARIMA, DNN, and LSTM models.

Before analysis, this study applied a lag of seven days between the input variables (optimal variable combination) and their associated output variable (disease occurrence). The OLS dataset was divided into a training data subset and a test data subset using a ratio of 8:2. This means all 569 rows of collected data were divided such that there were 455 rows for the training data subset and 114 rows for the test data subset. The training data subset was only used for model training. The test data subset was only used for prediction and performance evaluation in the model after training. The ARIMA dataset was also divided into a training data subset and test data subset using a ratio of 8:2, but only the disease occurrences were required for ARIMA. Similarly to the data above, the 569 rows of disease occurrence data were divided into 455 rows for the training data subset and 114 rows for the test data subset.

In the DNN and LSTM models, the whole dataset was divided into training, validation, and test data subsets at a ratio of 6:2:2 and training was performed. This means all 569 rows of collected data were divided into 341 rows for the training data subset, 114 rows for the validation data subset, and 114 rows for the test data subset. The training data subset was used for model training. The validation data subset was only used for performance evaluation during training. The final model after training was the model that yielded the best performance when the validation data subset was used in training. The test data subset was only used for the prediction and performance evaluation.

To compare the models, the root mean squared error (RMSE) was used to evaluate the prediction rates. RMSE is a common measurement for the difference between predicted and actual values. It is usually used in the other fields as well as in the prediction of infectious diseases [28,44,45]. RMSE is calculated using the equation below.

(1) $$\mathrm{RMSE}=\sqrt{\frac{\sum_{t=1}^{n}{\left(X_{t}-{\hat{X}}_{t}\right)}^{2}}{n}}\;$$

#### 2.2.1. Selecting Optimal Variable Combinations

The optimal variable combinations for the model were selected by considering all possible models in the regression analysis. The models are combinations of the four types of data in the dataset (Naver searches (n), Twitter searches (tw), temperature (t), and humidity (h)).

**Figure 2:**
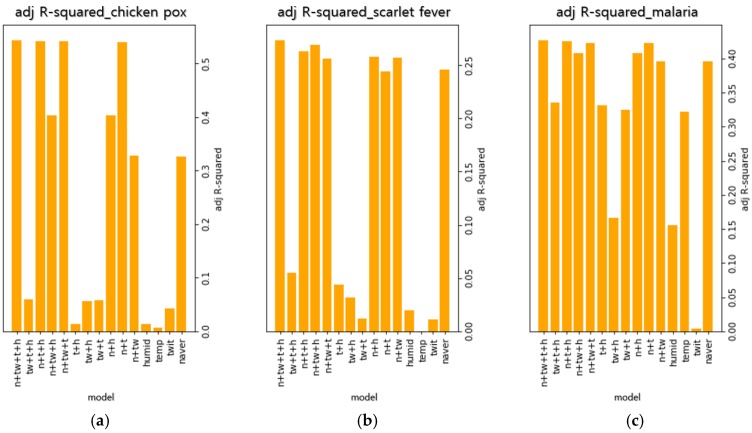
Variable optimization parameters. (**a**) Adjusted R-squared values of 15 regression models in chickenpox, (**b**) Adjusted R-squared values of 15 regression models in scarlet fever, (**c**) Adjusted R-squared values of 15 regression models in malaria.

Figure 2 shows the adjusted R-squared values of 15 regression models for each infectious disease. Among the observed regression models, the models that are combinations of all variables had the best explanatory power. Therefore, this combination was chosen as the optimal variable combination.

#### 2.2.2. Selecting the Optimal Prediction Time Difference

Previous results [43] have shown that it is possible to predict infectious disease at an early stage if a model is designed to consider the time difference between clinical data and non-clinical data. Based on this observation, our model was designed to consider the time difference in each data set. In this situation, “lag” refers to the time delay between the date the data is collected and the date at which the effects actually occur. This means analysis was performed by establishing the time difference between the four input variables used in this study and the output variable that is actually affected. For example, a lag of 1 means that the output variable of 2 January 2016 is calculated using the input variables of 1 January 2016.

**Figure 3:**
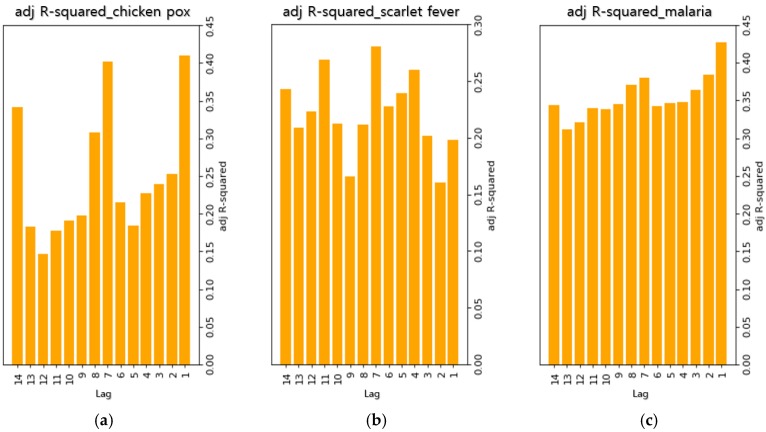
Adjusted R-squared for each lag. (**a**) Adjusted R-squared values of regression models that applied lag of 1–14 days in chickenpox, (**b**) Adjusted R-squared values of regression models that applied lag of 1–14 days in scarlet fever, (**c**) Adjusted R-squared values of regression models that applied lag of 1–14 days in malaria.

Figure 3 shows the adjusted R-squared values of regression models when 1–14 days of lag were tested for each of the infectious diseases in order to select the optimal lag. In the case of chickenpox, it was found that lags of 1, 7, and 14 days yielded the highest explanatory power. For scarlet fever, it was found that lags of 4, 7, and 11 days yielded the highest explanatory power. In the case of malaria, it was found that lags of 1, 2, and 7 days yielded the highest explanatory power. For chickenpox and malaria, the lag with the highest explanatory power was one day. However, it was decided that this lag was not suitable for the ultimate goal of reducing the length of delay from reporting to dissemination. In the observed regression models, the explanatory power of a lag of seven days was high for all infectious diseases. Therefore, it was decided that this lag was the most suitable and was used for later predictions.

#### 2.2.3. OLS

In this study, the OLS model was used to select the optimal parameter values. It was also used as a comparison model to evaluate the prediction performance of the deep learning models.

Linear regression is a regression analysis technique that models the linear correlation between the output variable *y* and one or more input variables *x* in the collected data. The model has the following form.

(2) $$y_{i}=\beta_{1}x_{i1}\;+\;\cdots \;+\beta_{p}x_{ip}+\varepsilon_{i}={\;x}_{i}^{T}\;\beta +{\;\varepsilon }_{i},\;i=1,\;\ldots ,\;n\;\;$$

OLS is the most simple and commonly used form of linear regression. It is a technique that minimizes the sum of squared errors and can solve the mathematical expression for ß, which is the parameter to be predicted, by using the equation below.

(3) $$\hat{\beta }={\left(X^{T}X\right)}^{-1}X^{T}y=\;{\left(\sum x_{i}x_{i}^{T}\right)}^{-1}\left(\sum x_{i}y_{i}\right)\;\;$$

OLS analyses were performed by R version 3.3.3 (https://www.r-project.org/).

#### 2.2.4. ARIMA

Because OLS is the simplest form of linear regression analysis, it is not sufficient for comparison with deep learning models. Therefore, we also compare the ARIMA model, which is often used for the prediction of infectious diseases [44,45,46]. This will more clearly compare traditional analysis methods (OLS and ARIMA) with deep learning (DNN and LSTM). The ARIMA model is a method for analyzing non-stationary time series data. One characteristic of ARIMA analysis is that it can be applied to any time series. In particular, it shows the detailed changes when the data fluctuates rapidly over time.

In this study, we used seasonal ARIMA because the collected data is seasonal. The seasonal ARIMA model is denoted as ARIMA(*p*, *d*, *q*)(*P*, *D*, *Q*)*_S_*. where *p* is the order of the autoregressive part, *d* is the order of the differencing, *q* is the order of the moving-average process, and *S* is the length of the seasonal cycle. (*P*, *D*, *Q*) is the seasonal part of the model. The seasonal ARIMA model is written below.

(4) $${\left(1-B\right)}^{d}{\left(1-B^{S}\right)}^{D}Y_{t}=\;\mu +\;\frac{\theta \left(B\right)\theta_{S}\left(B^{S}\right)}{\phi \left(B\right)\phi_{S}\left(B^{S}\right)}a_{t}\;$$

where $Y_{t}$ refers to the value of the time series at time t, μ is the mean term, $a_{t}$ is the independent disturbance, B is the backshift operator, ϕ(B) is the autoregressive operator, and θ(B) is the moving average operator. $\phi_{S}\left(B^{S}\right)\;$ and $\theta_{S}\left(B^{S}\right)$ are the seasonal operators of the model.

The ARIMA analyses were carried out using the R version 3.3.3.

#### 2.2.5. DNN

The DNN model is a feedforward analysis method that is a basic model for deep learning. DNN is composed of a minimum of three node layers and, with the exception of the input node, each node uses a nonlinear activation function. DNN uses a supervised learning technique called backpropagation. In this study, an infectious disease prediction model that uses DNN was designed and the basic DNN model was compared with this more advanced deep learning model.

The variables used in DNN are bias b, input x, output y, weight w, calculation function σ and activation function f(σ). Each neuron in DNN uses the following equation.

(5) σ :Sum=w·x+b 

(6) y :f(σ)=f(w·x+b) 

**Figure 4:**
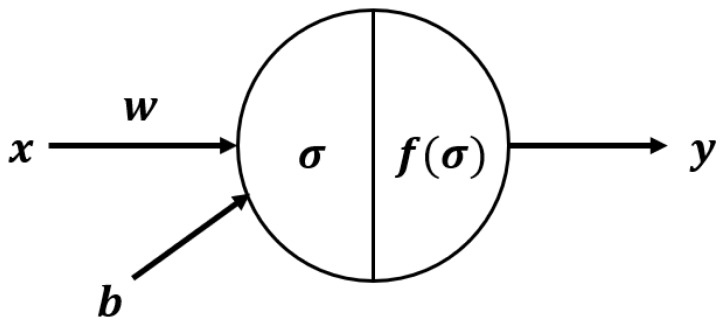
DNN structure. The variables used are bias b, input x, output y, weight w, calculation function σ and activation function f(σ).

Figure 4 shows the structure of a neuron in the DNN model. The DNN analyses were carried out using the “Dense layer” option of the Keras package in the Python version 3.5.3 (https://keras.io/). There are 10 parameters available in the Dense layer. We only modified the units, activation function, and dropout. The rest of the parameters used the default values (e.g., use_bias = True and kernel_regularizer = None).

#### 2.2.6. LSTM

The LSTM model is suitable for predicting time series data when there is a time step with a random size [47]. It was thought that prediction performance could be improved by creating an infectious disease prediction model using LSTM and the time series data collected in this study. 

An important advantage of recurrent neural networks (RNNs) is that contextual information is available when mapping IO sequences. However, there is a gradient problem in that the effect of a given input on the hidden layer can be increased or decreased significantly during the circular connection. As new inputs are overwritten, the sensitivity of the first input decreases over time. Therefore, the network is “forgotten”. The input gate, output gate, and forget gate are non-linear summation units that control the activation of the cell. The forget gate multiplies the previous state of the cell while the input and output gates multiply the IO of the cell. The activation function *f* of the gate is a logistic sigmoid. The IO activation functions *g* and *h* of the cell usually use hyperbolic tangents or logistic sigmoids. However, in some cases, *h* uses the identity function. As long as the forget gate is open and the input gate is closed, the memory cell continues to remember the first input. In this way, LSTM is an algorithm that resolves a problem in traditional RNNs [48].

The equations for forgetting, storing, renewing, and outputting information in the cell are shown below, respectively.

(7) $$f_{t}=\sigma \left(w_{f}\cdot \left[h_{t-1},\;x_{t}\right]+b_{f}\right)\;$$

(8) $$i_{t}=\sigma (W_{i}\cdot \left[h_{t-1},\;x_{t}\right]+b_{i})\;$$

(9) $${\tilde{C}}_{t}=tanh(W_{C}\cdot \left[h_{t-1},\;x_{t}\right]+b_{C})\;$$

(10) $$C_{t}=f_{t}\times \;C_{t-1\;}+\;i_{t}\times {\tilde{C}}_{t}\;\;$$

(11) $$o_{t}=\sigma (W_{o}\cdot \left[h_{t-1},\;x_{t}\right]+b_{o})\;$$

(12) $$h_{t}=o_{t}\times \tan h\left(C_{t\;}\right)\;$$

When data ($x_{t}$) is input to the LSTM cell in Equation (7), function $f_{t}$ determines the information to be forgotten in the cell layer. In Equations (8) and (9), information that will be newly saved in the cell layer is created in $i_{t}$ and ${\tilde{C}}_{t}$ In Equation (10), the cell layer $C_{t\;}$ is renewed using $f_{t}$, $i_{t}$, and ${\tilde{C}}_{t}$ In Equation (11), the cell layer’s information is used and $h_{t}$ is the output. In Equation (12), the cell state gets a value between −1 and 1 through the tanh function. The values of $C_{t\;}$ and $h_{t}$ are kept for the next iteration of LSTM. LSTM analyses were carried out using the “LSTM layer” of the Keras package in the Python version 3.5.3. There are 23 parameters available in the LSTM layer. We only set the units, activation function, return sequence, and dropout. The rest of the parameters used the default values (e.g., use_bias = True, recurrent regularizer = None, recurrent_constraint = None, and unit_forget_bias = None).

#### 2.2.7. Determining the Optimal Deep Learning Parameters

**Figure 5:**
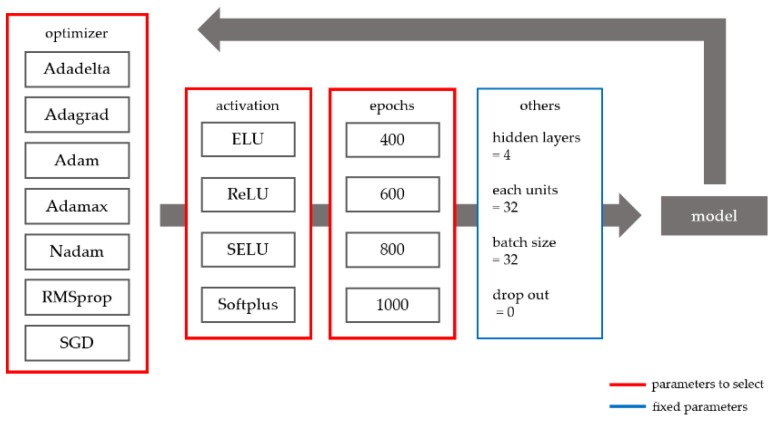
Deep learning parameter selection.

Figure 5 shows the parameter selection method for the deep learning approach used in this study. The Adadelta, Adagrad, Adam, Adamax, Nadam, RMSprop, and stochastic gradient descent (SGD) optimizers were compared. All parameters of each optimizer used the default values of the Keras package. For instance, in SGD, the learning late is 0.01, the momentum is 0, the decay is 0, and the Nesterov momentum is false. In addition, the following activation functions were evaluated: exponential linear unit (ELU), rectified linear unit (ReLU), scaled ELU (SELU), and Softplus. Lastly, various numbers of epochs (400, 600, 800, and 1000) were evaluated. The other parameters were fixed as follows: number of hidden layers = 4, number of units in each hidden layer = 32, batch size = 32, and drop out = 0. Prediction models with variable and fixed parameters were trained on the data and the resulting models were compared to determine the optimal prediction model. To ensure the amount of DNN model data was the same as that of the LSTM model, previous data from the same time period as the LSTM was inserted. All deep learning models were implemented using the Keras package in the Python version 3.5.3.

## 3. Results

### 3.1. OLS

The regression model was formed based on 569 days of data in which a lag of seven days was applied to each infectious disease dataset. The dataset was divided up in an 8:2 ratio and each part was used for constructing the regression model and prediction. Table 3 presents the OLS results.

Each regression model had results that were below the level of significance (*p* < 0.05). The adjusted R-squared value was greater than 0.25 for all three infectious diseases, which means the models can be said to have significant explanatory power. Of the infectious disease regression models, the chickenpox model yielded significant results for the Naver search queries, temperature, and humidity. The scarlet fever model yielded significant results for the Naver search queries and humidity. Additionally, the malaria model yielded significant results for the Naver search queries and temperature (*p* < 0.05). Looking at these results together, the Naver search query data was significant for all three infectious diseases and the Twitter data was not significant for any of the three. It can be seen that the Internet search query data can be used to design an infectious disease prediction model, which was reported by previous studies. However, the results for the Twitter data differ from the results of previous studies. This is believed to be because Naver accounted for the largest share (86.2%) of Korean search engine use in the health/medicine field for the first half of 2017 while Twitter accounted for the smallest share (0.5%) of social media use in the health/medicine field for the same time period (http://internettrend.co.kr/trendForward.tsp). However, the Twitter data had an effect on the process of finding the model with the highest adjusted R-squared value. Therefore, it is expected to have an effect on future analysis as well. The temperature had a significant relationship with all infectious diseases except for scarlet fever and humidity had a significant relationship with all infectious diseases except for malaria. The values of the coefficients show that the most significant variables for chickenpox and scarlet fever was the Naver search query data (4.4589 and 2.1956, respectively) and, for malaria, it was the temperature values (0.0770). The effect of Naver search query data in particular was significant for all three infectious diseases, which confirms that it can be suitable for predicting infectious disease.

### 3.2. ARIMA

The seasonal ARIMA model was evaluated using the same data used for OLS. The autocorrelation function and the partial autocorrelation function were checked for the seasonality of infectious diseases and seasonality was observed. It was considered inappropriate to select the parameters (e.g., *p*, *d*, *q*) because cuts off and tails off are unclear. Therefore, the optimal model for each infectious disease was selected based on the Akaike information criterion (AIC) and RMSE. The AIC and RMSE were used to compare the ARIMA models.

Table 4 shows the AIC and RMSE of the seasonal ARIMA model for each infectious disease, which allows us to identify the top three ARIMA model. In addition, the choice of parameter values did not substantially affect the AIC and RMSE of the model for a single infectious disease.

### 3.3. Top-Ranked DNN and LSTM Prediction Models

**Figure 6:**
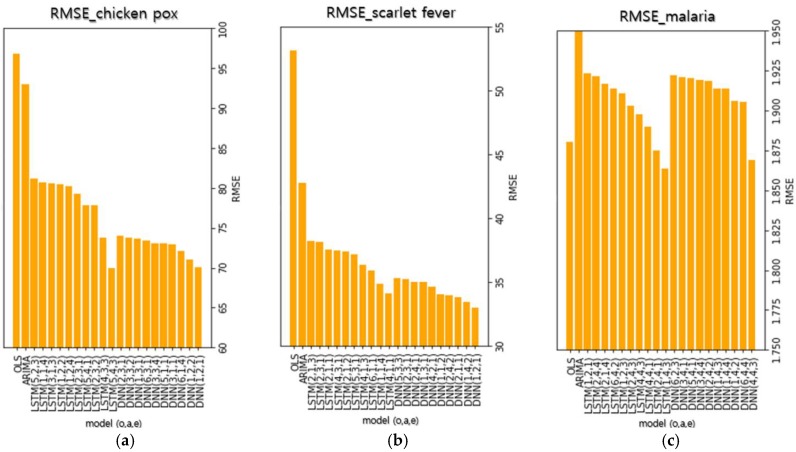
Ten models with the lowest RMSEs for each infectious disease. The numbers in parentheses indicate each model’s optimizer, activation, and number of epochs, respectively. (optimizer) 1: Adadelta, 2: Adagrad, 3: Adam, 4: Adamax, 5: Nadam, 6: RMSProp, and 7: SGD, (activation function) 1: ELU, 2: ReLU, 3: SELU, and 4: SoftPlus, (number of epochs) 1: 400, 2: 600, 3: 800, and 4: 1000. (**a**) The top-10 deep learning models and traditional analysis models with the lowest RMSEs in chickenpox, (**b**) The top-10 deep learning models and traditional analysis models with the lowest RMSEs in scarlet fever, (**c**) The top-10 deep learning models and traditional analysis models with the lowest RMSEs in malaria.

To compare the performance of each model, Figure 6 shows the 10 models with the lowest RMSE of the test data subset. The numbers inside the parentheses in each model’s name represent the optimizer, the activation function, and the number of epochs used in the models (e.g., DNN (1, 2, 3) indicates that the optimizer, the activation function, and the number of epoch are Adadelta, ReLU, and 800, respectively). The metric used to compare the models is RMSE, which shows the difference between the actual and predicted values. A smaller RMSE value indicates a smaller difference between the actual and predicted values and indicates a higher prediction performance.

Table S1 shows the RMSE and prediction graphs of the DNN and LSTM models with the lowest RMSE for chickenpox. It can be seen that the prediction graphs for each analysis method have similar shapes overall. The 10 DNN models for chickenpox had a mean RMSE of 72.8215 and a standard deviation of 1.28, which shows stable model performance. When the prediction performances of each model were compared based on RMSE, the top 10 DNN models showed a 24.45% performance improvement compared to the ARIMA model. The mean RMSE of the 10 LSTM models was 78.2850, which is higher than the DNN models. The standard deviation was 3.64, which shows that the difference among LSTM models was more marked than the difference among DNN models. Despite this, the top 10 LSTM models achieved an 18.78% performance improvement over the ARIMA model on average. There was a difference between the DNN and LSTM models’ average figures and standard deviations. However, in the models with the lowest RMSE for each analysis method, there was not a big difference, which indicates that there was not a large difference in performance when the optimal parameters for each analysis method were used. Table S2 shows the RMSE and the prediction graphs of the DNN and LSTM models with the lowest RMSE for scarlet fever. The shapes of the graphs for the DNN models are similar. For the LSTM models, the shapes of the graphs are similar except for the model with the lowest RMSE. Unlike the graphs of the other LSTM models, the graph LSTM model with the lowest RMSE showed a strong tendency to follow the actual trend. This result infers that a prediction model that is better than existing prediction models can be designed by changing the deep learning parameters to achieve optimization. As is the case with chickenpox, the mean RMSE of the DNN model (34.4347) for scarlet fever was lower than that of the LSTM model (36.8140). The standard deviation of the DNN model (0.80) was also lower than that of the LSTM model (1.37). When comparing each model based on RMSE, the top 10 DNN models showed a 23.28% performance improvement over the ARIMA model and the LSTM models showed a 17.97% performance improvement over the ARIMA model. Table S3 shows the RMSE and the prediction graphs of the DNN and LSTM models with the lowest RMSE for malaria. Like other infectious diseases, the DNN model prediction graphs have a similar shape. However, the shapes of the LSTM model prediction graphs have a tendency to not follow the trend. The RMSEs of each prediction model, excluding the ARIMA model, showed little difference. This is believed to be because the number of malaria occurrences is fewer than those of the other infectious diseases. Therefore, adequate predictions could not be formed.

### 3.4. Optimal Performance Model

It is difficult to understand the special characteristics of each analysis method by simply comparing RMSE figures alone. Therefore, a detailed comparison was performed on the basic comparison models (OLS and ARIMA) and the analysis methods that use deep learning (DNN and LSTM). The deep learning models used for comparison were the models with the optimal performance and lowest RMSE so that they could best represent each analysis method. With the best performance.

**Figure 7:**
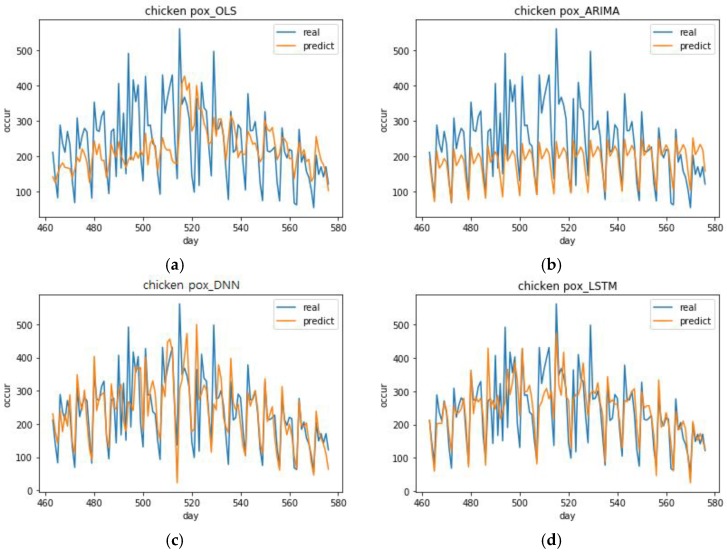
Top performing chickenpox predictions for each analysis method. (**a**) Prediction graph of OLS model in chickenpox, (**b**) Prediction graph of ARIMA model in chickenpox, (**c**) Prediction graph of DNN (1, 2, 1) model with the best performance in chickenpox, (**d**) Prediction graph of LSTM (5, 4, 3) model with the best performance in chickenpox.

Figure 7 shows the chickenpox predictions of the model with the lowest RMSE out of the 10 models with the lowest RMSE for each analysis method. The DNN model with the best performance had the following specifications, which include optimizer = Adadelta, activation function = ReLU, and number of epochs = 400 (DNN (1, 2, 1)). The LSTM model with the best performance had the following specifications, which include optimizer = Nadam, activation function = SoftPlus, epochs = 800 (LSTM (5, 4, 3)). The OLS model’s predictions had a smaller range of fluctuation than the deep learning models. From day 480, it seems to follow the trend, but it does not follow the small changes. After day 550, it cannot predict the downward shape even within a stable graph model. In short, the OLS model is not suitable as a prediction model. The ARIMA model’s prediction graph has a very simple shape. This model is cyclic and there is a slight increasing trend in which the predicted value per cycle increases by a factor of about 2.5 each time. This model cannot predict the trend at all. It can only predict a stable cyclic behavior. In contrast, the DNN (1, 2, 1) predictions followed the actual occurrence trend well. Moreover, it had a large range of fluctuation, which means it made accurate predictions overall. However, when the number of occurrences rose rapidly in days 510–520, it was unable to follow these values. The LSTM (5, 4, 3) predictions had a smaller range of fluctuation than the DNN (1, 2, 1) model. Its range of variance was small, which means it had a stable shape and it performed better than DNN (1, 2, 1) when the number of occurrences rose rapidly.

**Figure 8:**
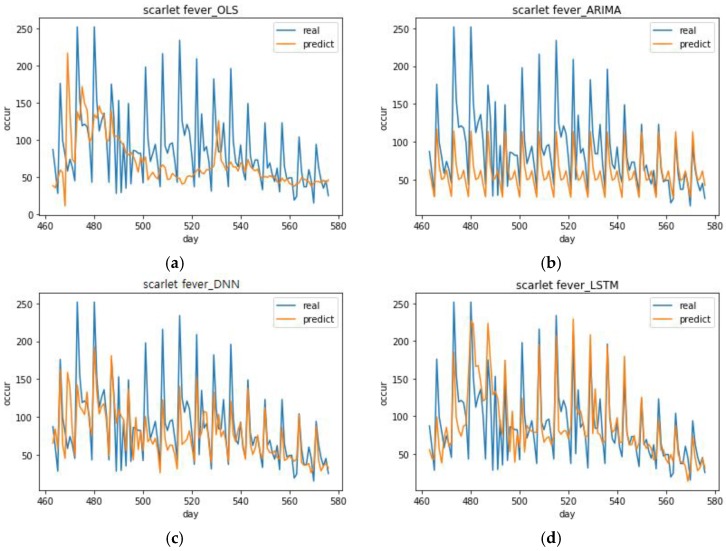
Top performing scarlet fever predictions for each analysis method. (**a**) Prediction graph of OLS model in scarlet fever, (**b**) Prediction graph of ARIMA model in scarlet fever, (**c**) Prediction graph of DNN (1, 1, 2) model with the best performance in scarlet fever, (**d**) Prediction graph of LSTM (4, 1, 1) model with the best performance in scarlet fever.

Figure 8 shows the scarlet fever predictions of the models with the best performance for each analysis method. The DNN model with the best performance had the following specifications: optimizer = Adadelta, activation function = ELU, and number of epochs = 600 (DNN (1, 1, 2)). The LSTM model with the best performance had the following specifications: optimizer = Adamax, activation function = ELU, number of epochs = 400 (LSTM (4, 1, 1)). The OLS model’s predictions were completely unable to follow the trend, which was similar with the chickenpox case. The ARIMA model’s prediction has no particular merit because it also predicts a simple cycle. Much like its chickenpox prediction, it can only predict a stable cyclic behavior. The DNN (1, 1, 2) predictions were relatively close when the number of occurrences was low, but they were too low when the number of occurrences was high. LSTM (4, 1, 1) had a larger range of variance than DNN (1, 1, 2) and its predictions were close when the number of occurrences was high. In the prediction graphs for all of the top performing scarlet fever models, none of the models were able to follow the trend on days 480–500 when there was a severe variance in the number of actual occurrences. Looking at the mean of each model’s predicted value for the number of occurrences, the LSTM model (88.7568) was larger than the mean of the DNN model (77.096). These same results were also seen in the case of chickenpox (DNN model mean = 237.5318, LSTM model mean = 241.5186). This showed that more suitable results can be obtained if the LSTM model is used to predict the maximum value for the number of occurrences and the DNN model is used to predict the minimum value.

**Figure 9:**
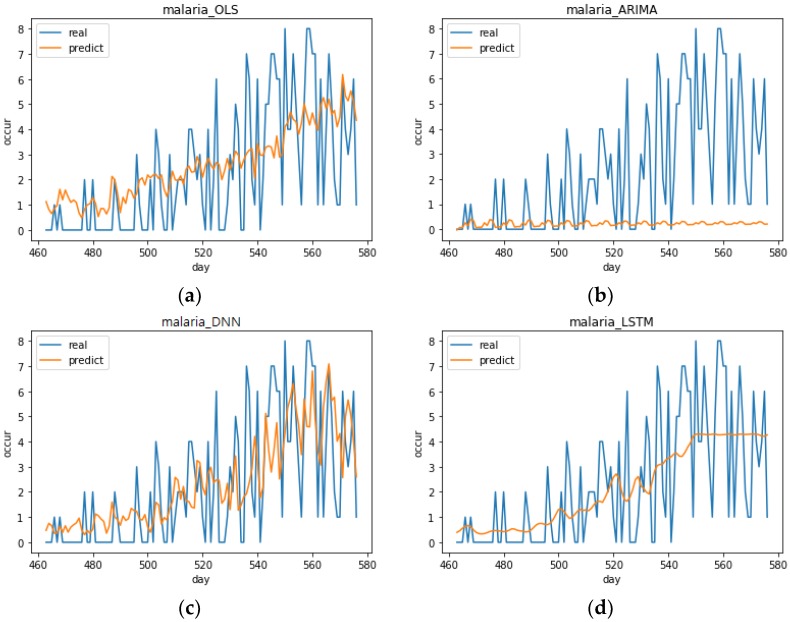
Top performing malaria predictions for each analysis method. (**a**) Prediction graph of OLS model in malaria, (**b**) Prediction graph of ARIMA model in malaria, (**c**) Prediction graph of DNN (4, 4, 3) model with the best performance in malaria, (**d**) Prediction graph of LSTM (1, 4, 3) model with the best performance in malaria.

Figure 9 shows the malaria predictions of the models with the best performance for each analysis method. The DNN model with the best performance had the following specifications: optimizer = Adamax, activation function = SoftPlus, number of epochs = 800 (DNN (4, 4, 3)). The LSTM model with the best performance had the following specifications: optimizer = Adadelta, activation function = SoftPlus, number of epochs = 800 (LSTM (1, 4, 3)). The predictions of the analysis methods were not satisfactory, but the DNN (4, 4, 3) model’s predictions seemed to follow the trend relatively well. The ARIMA model predicts values close to 0. It is believed that the occurrences in the malaria data are less than those of other diseases and, therefore, not suited to time series analysis because occurrences are concentrated in the summer seasons. As seen in Section 3.3, the lowest RMSEs of each prediction model excluding the ARIMA model showed little difference. LSTM (1, 4, 3) had a lower range of variance than OLS and it seemed completely unable to make predictions. As mentioned before, the reason that predictions were inadequate for all of the models in addition to LSTM (1, 4, 3) was that the number of malaria occurrences was small and proper results could not be produced.

When the predictions were compared according to each analysis method, the DNN and LSTM deep learning models performed better than the OLS and ARIMA models by assuming that there was a sufficiently large number of occurrences. When comparing the DNN and LSTM models, the best models had similar performance, but the DNN models were better in terms of average performance. However, when the number of occurrences was large, the LSTM model made close predictions. It seems to be an analysis method that is suitable for circumstances when the number of occurrences is rapidly increasing and infectious disease is believed to be spreading.

## 4. Discussion

The deep learning model showed outstanding performance compared to the traditional ARIMA method. Of all the DNN and LSTM prediction models for chickenpox, the optimal models with the lowest RMSE yielded 27.22% and 27.33% better performance than the ARIMA model, respectively. The top 10 DNN models for chickenpox improved performance by an average of 24.45% and the LSTM models improved performance by an average of 18.78%. The lowest RMSEs of the DNN and LSTM prediction models for scarlet fever showed 26.25% and 23.79% improved performances compared to ARIMA models. The top 10 DNN models for scarlet fever improved performance by an average of 23.28%. The LSTM models improved performance by an average of 17.97%.

As noted in the previous sections, it was difficult to predict infectious diseases when the number of infections was small and concentrated in one season. In effect, we observed that the incidence of malaria was high over days 160–250 and after day 530. This period corresponds to the summer season in Korea. Predicting infectious diseases with this particular data set was difficult and it was not suitable for the ARIMA analysis. Even using this particular data set, when DNN was used, the trend of infectious diseases was followed comparatively (Figure 9). Moreover, there is a possibility that the performance would be improved in the DNN model if more diverse parameters were adjusted. This means using deep learning has the advantage of scalability and this can be further investigated in future studies.

The ARIMA model that was used in this study was observed to be effective if the number of incidences of infectious diseases was regular and had no increasing or decreasing trends. However, actual data can have trends and be irregular. Therefore, deep learning can be an excellent analytical method when analyzing such data and predicting future situations. According to the results of the previous analyses, the deep learning model follows increasing and decreasing trends sufficiently well. Moreover, the DNN and LSTM models were observed to be sensitive to decreasing trends and increasing trends, respectively.

## 5. Conclusions

Infectious disease is a social problem in that it can cause not only personal damage but also widespread harm. For this reason, research is being conducted to minimize social losses by predicting the spread of infectious diseases. The aim of this study was to design an infectious disease prediction model that is more suitable than existing models by using various input variables and deep learning techniques. Therefore, in this study, the optimal parameters were set using a variable selection method based on OLS. The relationship between actual instances of disease occurrence and the Internet search query data tends to have a time lag, which means a lag was added to each infectious disease’s dataset to find the future trend. Next, an analysis of ARIMA, DNN, and LSTM was performed with optimal parameters.

The results of OLS analysis using optimal parameters showed that the regression models for each infectious disease had significant results. Of the four input variables, the Naver search frequency had a significant relationship with all three infectious diseases. The performance of the OLS and ARIMA analysis was used to evaluate the deep learning models. Looking at the results for DNN and LSTM, both the deep learning models made much better predictions than the OLS and ARIMA models for all infectious diseases. Moreover, the DNN models had the best performance on average, but the LSTM models made more accurate predictions when infectious diseases were spreading. However, in the case of malaria, there were few occurrences of the disease compared to other infectious diseases, which means the predictions were not comparatively accurate.

This study was also able to reveal special characteristics of the DNN and LSTM models. The DNN model produced smaller values than the LSTM model on average when predicting infectious diseases. Suitable predictions can be made using the DNN model when predicting the minimum value for disease occurrence and using the LSTM model when predicting the maximum value.

In previous studies, deep learning algorithms were not used [10,11,12,13,14,15,17] or the amount of data considered was small [22,23,28,29]. This study used social media big data and weather data, which have not been sufficiently considered in existing studies. It also used deep learning analysis, which yields high prediction performance to increase the performance of infectious disease predictions. The results showed that, when selecting the optimal parameters, adding all input variables had the highest explanatory power. This means that, by adding various data, it was possible to design a model with higher explanatory power. Moreover, the LSTM model results for scarlet fever indicate that it is possible to optimize a deep learning model by changing its parameters in various ways and, therefore, design a prediction model that is better than existing prediction models.

This study has reviewed the factors involved with infectious disease occurrence using search query data and social media big data, which exist because of the development of the Internet as well as temperature and humidity weather data. It also constructed traditional prediction models such as OLS, ARIMA, and deep learning prediction models such as DNN and LSTM and compared their prediction performance to confirm that the models that use deep learning are the most suitable for infectious disease prediction. It is believed that infectious disease prediction models that employ deep learning can be used to supplement current infectious disease surveillance systems and, at the same time, predict trends in infectious disease. If this can reduce the time differences in reporting systems so that infectious disease trends can be known immediately, it is expected that immediate responses to infectious disease will become possible and costs to society can be minimized. According to a study by Shin et al., an emerging infectious disease known as the Middle East Respiratory Syndrome (MERS) has a deep correlation with Internet search data [20] and it will become possible to expand these methods to the real-time surveillance and prediction of emerging infectious diseases as well.

However, this study has three limitations, which include a relatively short data collection period, regionally combined predictions, and a consideration of a narrow range of parameters in the deep learning model. The search query data collection time period used in this research was relatively short extending from 1 January 2016 to 29 July 2017. The particular spatial ranges of data were averaged across the whole of South Korea. It is believed that, if the data is expanded and the spatial ranges are subdivided, the model’s performance will improve. In addition, an effort was made to change the DNN and LSTM model parameters and create a variety of prediction models, but the deep learning prediction models used in this study did not cover all the prediction models that could be implemented. Parameters such as hidden layers and batch size were not considered. Therefore, it is difficult to conclude that the most effective model was created. If more parameters are considered and more prediction models are made in future research, it is believed that prediction performance can be increased somewhat.

## Figures and Tables

**Table 1:** Description of data types. KCDC: Korea Center for Disease Control; KMA: Korea Meteorological Administration’s weather information open portal.

Variable	Source	Description	Number of Observations
**Occurrences**	KCDC	Daily number of confirmed infectious disease diagnoses	576
**Naver**	Naver Data Lab	Daily Naver search frequency	
**Twitter**	Twitter	Daily number of Twitter mentions	
**Temperature**	KMA	Average daily temperature for all of South Korea	
**Humidity**		Average daily humidity for all of South Korea	

**Table 2:** Data statistics.

Disease	Variable	Min.	Median	Mean	Max.	Var.	SD
**Chicken Pox**	Occurrences	16	146.5	166.76	562	9676.29	98.37
	Naver	10.56	29.01	33.94	100	239.97	15.50
	Twitter	1	10	12.81	194	220.39	14.85
**Scarlet fever**	Occurrences	3	37	46.33	252	1346.85	36.70
	Naver	0.32	2.72	4.99	100	72.63	8.52
	Twitter	0	0	0.27	15	1.06	1.03
**Malaria**	Occurrences	0	0	1.65	14	6.65	2.58
	Naver	5.52	17.24	23.13	100	193.02	13.89
	Twitter	0	3	4.23	34	15.08	3.88
**Environ-mental variables**	Temperature (°C)	−10.82	14.19	13.27	29.54	94.07	9.70
	Humidity (%)	30.87	67.31	66.70	94.31	172.81	13.15

**Table 3:** OLS Results.

Disease	$R^{2}$	$\mathrm{Adjusted}\;R^{2}$	*F*	*p*	Variable	Coefficient	*T*	*p*
Chickenpox	0.4077	0.4035	97.0659	<0.001	Naver	4.4569	18.2096	<0.001
					Twitter	0.2162	0.9769	0.3290
					Temperature	−3.8421	−8.7717	<0.001
					Humidity	−0.8919	−3.1027	0.0020
					Intercept	121.9521	6.6302	<0.001
Scarlet fever	0.2867	0.2817	56.6851	<0.001	Naver	2.1956	12.6929	<0.001
					Twitter	−1.981	−1.3940	0.1639
					Temperature	0.2559	1.5660	0.1179
					Humidity	−0.5369	−4.4766	0.0491
					Intercept	68.5623	9.4170	<0.001
Malaria	0.3863	0.3819	88.7462	<0.001	Naver	0.0649	7.1140	<0.001
					Twitter	0.0369	1.6594	0.0976
					Temperature	0.0770	5.7623	<0.001
					Humidity	0.0129	1.6421	0.1011
					Intercept	−1.8257	−3.7883	<0.001

**Table 4:** Results of seasonal ARIMA. AIC: Akaike information criterion; RMSE: root mean squared error.

Disease	Models	AIC	RMSE
Malaria	$\mathrm{ARIMA}\left(1,\;1,\;1\right){\left(1,\;0,\;1\right)}_{7}$	1740.807	3.179
	$\mathrm{ARIMA}\left(2,\;1,\;2\right){\left(1,\;0,\;1\right)}_{7}$	1741.823	3.174
	$\mathrm{ARIMA}\left(1,\;1,\;1\right){\left(2,\;0,\;1\right)}_{7}$	1742.746	3.179
Scarlet fever	$\mathrm{ARIMA}\left(1,\;0,\;1\right){\left(0,\;1,\;2\right)}_{7}$	3513.540	44.884
	$\mathrm{ARIMA}\left(1,\;0,\;2\right){\left(0,\;1,\;2\right)}_{7}$	3513.891	45.340
	$\mathrm{ARIMA}\left(2,\;0,\;2\right){\left(1,\;1,\;1\right)}_{7}$	3515.127	46.026
Chickenpox	$\mathrm{ARIMA}\left(1,\;0,\;4\right){\left(0,\;1,\;2\right)}_{7}$	4576.544	96.398
	$\mathrm{ARIMA}\left(2,\;0,\;4\right){\left(1,\;1,\;2\right)}_{7}$	4577.808	97.256
	$\mathrm{ARIMA}\left(2,\;0,\;2\right){\left(1,\;1,\;1\right)}_{7}$	4583.896	96.835

## Supplementary Materials

The following are available online at http://www.mdpi.com/1660-4601/15/8/1596/s1, Table S1: The root mean squared error (RMSE) and prediction graphs of top 10 deep neural network (DNN) and long-short term memory (LSTM) models for chickenpox. The seasonal autoregressive integrated moving average (ARIMA) model is denoted as ARIMA(*p*, *d*, *q*)(*P*, *D*, *Q*)*_S_*. where *p* is the order of the autoregressive part, *d* is the order of the differencing, *q* is the order of the moving-average process, and *S* is the length of the seasonal cycle. (*P*, *D*, *Q*) is the seasonal part of the model. The numbers in parentheses indicate each deep learning model’s optimizer, activation, and number of epochs, respectively. (optimizer) 1: Adadelta, 2: Adagrad, 3: Adam, 4: Adamax, 5: Nadam, 6: RMSProp, and 7: SGD, (activation function) 1: ELU, 2: ReLU, 3: SELU, and 4: SoftPlus, (number of epochs) 1: 400, 2: 600, 3: 800, and 4: 1000, Table S2: The RMSE and prediction graphs of the top 10 DNN and LSTM models for scarlet fever, Table S3: The RMSE and prediction graphs of top 10 DNN and LSTM models for malaria.
