# Supplementary material for: Predicting Infectious Disease Using Deep Learning and Big Data
Source: Int J Environ Res Public Health. 2018 Jul 27;15(8):1596. doi: 10.3390/ijerph15081596 (PMC6121625; doi:10.3390/ijerph15081596)
Supplement: Supplementary file 1 [file ijerph-15-01596-s001.pdf]

Table S1. The root mean squared error (RMSE) and prediction graphs of top 10 deep neural network (DNN) and long-short term memory (LSTM) models for chickenpox. The seasonal autoregressive integrated moving average (ARIMA) model is denoted as ARIMA(p, d, q)(P, D, Q)$_S$. where p is the order of the autoregressive part, d is the order of the differencing, q is the order of the moving-average process, and S is the length of the seasonal cycle. (P, D, Q) is the seasonal part of the model. The numbers in parentheses indicate each deep learning model's optimizer, activation, and number of epochs, respectively. (optimizer) 1: Adadelta, 2: Adagrad, 3: Adam, 4: Adamax, 5: Nadam, 6: RMSProp, and 7: SGD, (activation function) 1: ELU, 2: ReLU, 3: SELU, and 4: SoftPlus, (number of epochs) 1: 400, 2: 600, 3: 800, and 4: 1000.

**Ordinary least squares**			**ARIMA$(1,0,4)(0,1,2)_7$**		
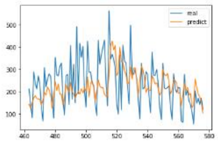			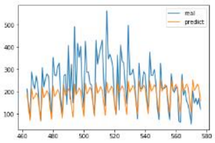		
**DNN**			**LSTM**		
Model(o, a, e)	RMSE	Prediction	Model(o, a, e)	RMSE	Prediction
DNN(2, 3, 1)	74.1217	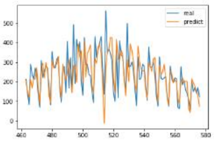	LSTM(5, 2, 3)	81.3131	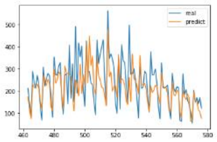
DNN(3, 3, 2)	73.9389	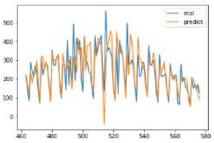	LSTM(1, 1, 4)	80.7757	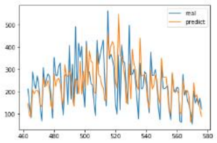
DNN(1, 3, 1)	73.7468	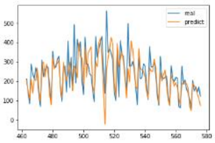	LSTM(3, 1, 3)	80.7138	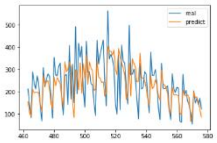
DNN(6, 3, 1)	73.4877	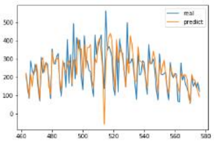	LSTM(1, 2, 2)	80.5656	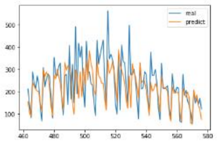
DNN(3, 3, 4)	73.1942	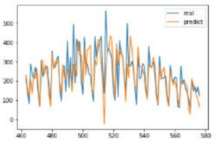	LSTM(1, 2, 4)	80.3162	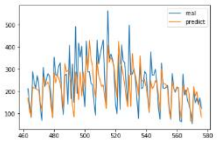
DNN(5, 1, 1)	73.1822	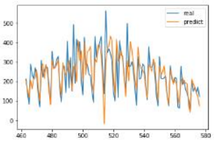	LSTM(2, 3, 1)	79.3532	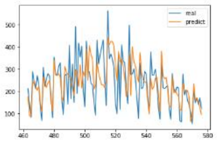
DNN(3, 1, 1)	73.0427	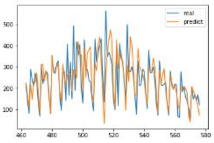	LSTM(5, 4, 1)	77.9777	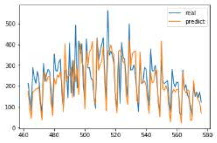
DNN(6, 1, 4)	72.1856	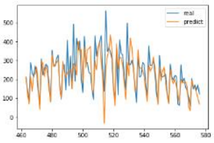	LSTM(2, 3, 2)	77.8980	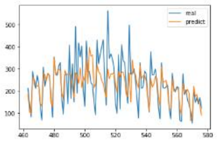

DNN(1, 2, 2)	71.1555	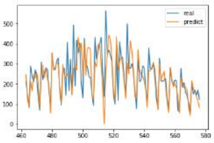	LSTM(4, 3, 3)	73.8884	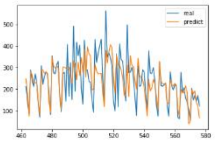
DNN(1, 2, 1)	70.1595	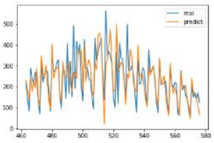	LSTM(5, 4, 3)	70.0479	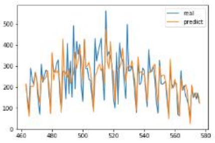

Table S2. The RMSE and prediction graphs of top 10 DNN and LSTM models for scarlet fever.

**Ordinary least squares**			**ARIMA$(1, 0, 1)(0, 1, 2)_7$**		
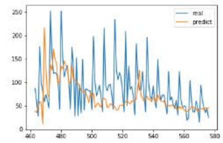			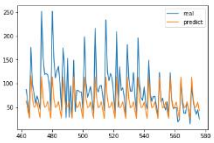		
**DNN**			**LSTM**		
Model(o,a,e)	RMSE	Prediction	Model(o,a,e)	RMSE	Prediction
DNN(5, 3, 3)	35.3867	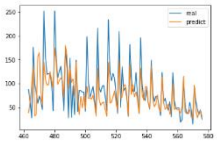	LSTM(2, 1, 3)	38.3012	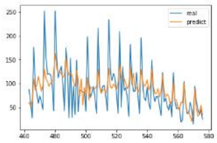
DNN(2, 1, 3)	35.3123	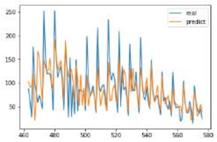	LSTM(2, 3, 1)	38.2312	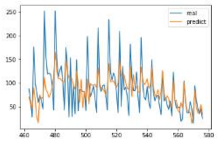
DNN(2, 1, 4)	35.1069	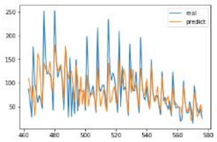	LSTM(2, 1, 1)	37.6151	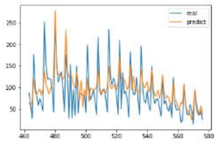
DNN(1, 1, 3)	35.0984	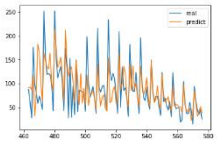	LSTM(4, 3, 1)	37.5653	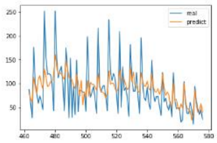
DNN(4, 1, 2)	34.6905	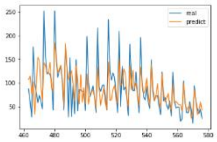	LSTM(2, 1, 2)	37.5128	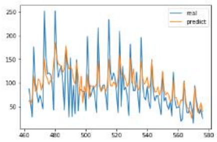
DNN(1, 2, 1)	34.1304	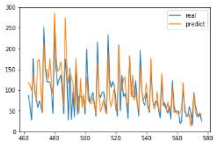	LSTM(5, 3, 1)	37.2380	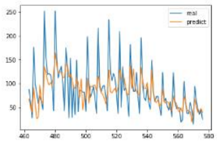
DNN(2, 2, 4)	34.0594	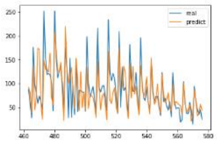	LSTM(4, 1, 3)	36.4692	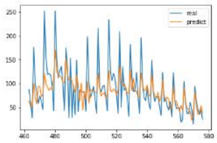
DNN(2, 1, 1)	33.9251	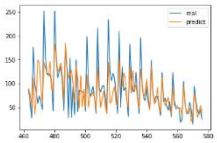	LSTM(6, 1, 1)	36.0120	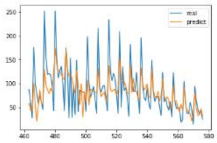
DNN(1, 2, 4)	33.5335	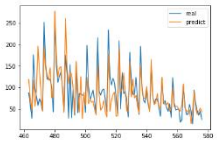	LSTM(1, 1, 4)	34.9879	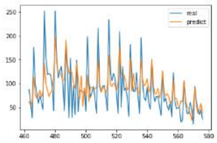
DNN(1, 1, 2)	33.1039	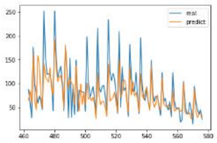	LSTM(4, 1, 1)	34.2074	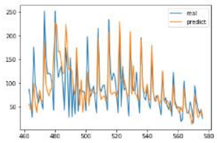

Table S3. The RMSE and prediction graphs of top 10 DNN and LSTM models for malaria.

**Ordinary least squares**			**ARIMA$(1,1,1)(1,0,1)_7$**		
	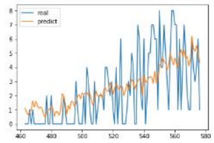			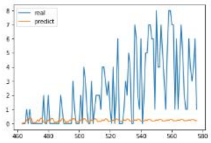	
	**DNN**			**LSTM**	
Model(o,a,e)	RMSE	Prediction	Model(o,a,e)	RMSE	Prediction
DNN(6, 2, 3)	1.9228	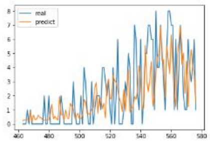	LSTM(1, 2, 1)	1.9240	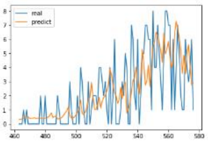
DNN(3, 4, 1)	1.9216	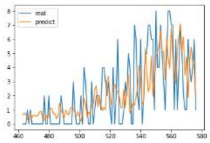	LSTM(2, 4, 4)	1.9219	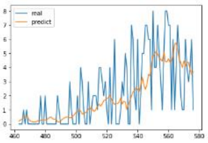
DNN(5, 4, 1)	1.9212	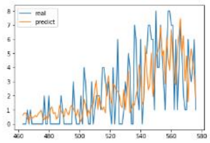	LSTM(2, 1, 4)	1.9174	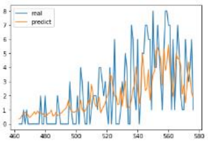
DNN(3, 4, 4)	1.9200	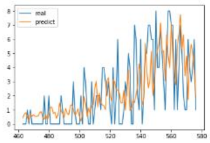	LSTM(6, 4, 2)	1.9143	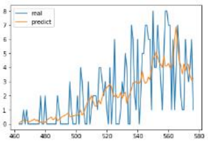
DNN(2, 4, 2)	1.9193	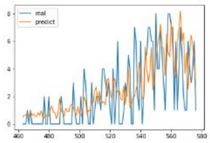	LSTM(1, 2, 3)	1.9113	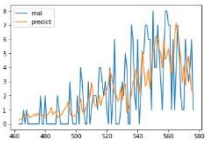
DNN(1, 4, 3)	1.9146	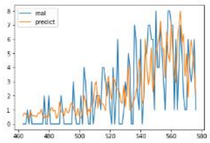	LSTM(2, 4, 3)	1.9034	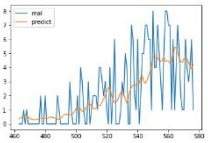
DNN(1, 4, 4)	1.9143	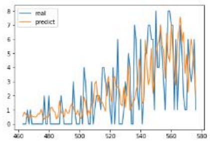	LSTM(4, 4, 3)	1.8986	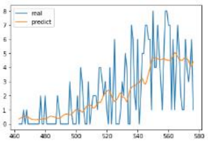
DNN(1, 4, 2)	1.9070	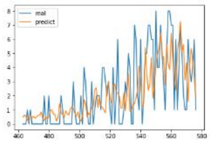	LSTM(4, 4, 1)	1.890	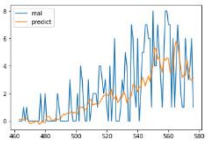
DNN(6, 4, 4)	1.9060	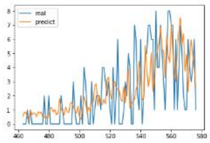	LSTM(2, 4, 1)	1.8754	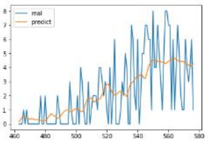
DNN(4, 4, 3)	1.8699	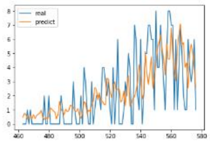	LSTM(1, 4, 3)	1.8641	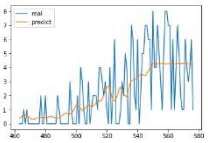
